# Supplementary material for: Feasibility of Community Pharmacist-Initiated and Point-of-Care CYP2C19 Genotype-Guided De-Escalation of Oral P2Y12 Inhibitors
Source: Genes (Basel). 2023 Feb 25;14(3):578. doi: 10.3390/genes14030578 (PMC10048116; doi:10.3390/genes14030578)
Supplement: Supplementary file 1 [file genes-14-00578-s001.zip › Supplementary File S1 Cardiologist letter and survey.pdf]

Leids Universitair Medisch Centrum

CYP2C19 GENOTYPING OF PATIENTS ON PRASUGREL/TICAGRELOR THERAPY

Study on the feasibility of switching prasugrel or ticagrelor to clopidogrel based on CYP2C19 genotype status, using a point-of- care CYP2C19 device in community pharmacy

Cardiologist Survey CYP2C19 Feasibility V1

Introduction

Dear Colleague,

You are receiving this email because a patient of yours is participating in the CYP2C19 feasibility study, a study on the feasibility of converting ticagrelor or prasugrel to clopidogrel in the community pharmacy based on the CYP2C19 genotype status. Your patient's pharmacist may have contacted you before regarding the CYP2C19 test results and eligible switch to clopidogrel. We would like to know your view on pharmacogenetic testing and how the application of and communication about the test results were experienced. The purpose of the questionnaire is to evaluate these experiences and identify potential barriers to pharmacogenetic testing in the community pharmacy setting.

Purpose of the questionnaire

It is important for us to know how the CYP2C19 test results and the pharmacist's recommendations are experienced by you as a treating physician. The purpose of this questionnaire is to assess these experiences and identify possible areas for improvement.

Confidentiality of data

Your answers will be kept confidential. No one except the researchers will have access to personal data and answers. By completing this questionnaire, you agree to participate in this study.

The survey will take a total of about **4-5 minutes** to complete.

Thank you in advance for your cooperation!

Questions

Do you have any questions regarding the study? If so, please contact the researchers:

Dr. Melina Den Haan (Cardiologist LUMC) : [e-mail-address]
Amar Levens MSc (Pharmacist-researcher LUMC): [e-mail-address] and [phone number]

**What is your age?**
…………….. years

**What is your gender?**
Male
Female

**What is your current position? And in which department are you employed?**	
1	Medical specialist, department…….
2	Medical specialist in training, department…….
3	Doctor not in training, department…….
4	Other, namely: , department…….

**How long have you been working as a physician?**
…………… years

**Can you indicate your level of knowledge regarding pharmacogenetics? (Pharmacogenetics examines the relationship between variations in DNA and individual response to drugs)**	
0 = no knowledge at all	10 = complete knowledge
0 1 2 3 4 5 6 7 8 9 10	

**Has pharmacogenetic testing ever been performed on your initiative?**	
1	Yes
2	No

**How often have you seen pharmacogenetic test results from patients so far?**	
1	More than 10 patients
2	6 to 10 patients
3	1 to 5 patients
4	This is the first time

**Statement: I think pharmacogenetics has added value in practice. *Can you elaborate on this?***	
1	Completely disagree
2	Disagree
3	Don't agree, don't disagree
4	Agree

5	Completely agree
Explanation:	

Statement: I need more support to apply pharmacogenetics in practice. *Can you elaborate on this?*	
1	Completely disagree
2	Disagree
3	Don't agree, don't disagree
4	Agree
5	Completely agree
Explanation:	

Statement: I do not understand what the pharmacogenetic test result means for the care I provide*? Can you elaborate on this?*	
1	Completely disagree
2	Disagree
3	Don't agree, don't disagree
4	Agree
5	Completely agree
Expalantion:	

Do you currently have any unanswered questions about the results of the pharmacogenetic test? *Can you elaborate on this?*	
1	Yes
2	No
Explanation:	

**In your opinion, who is the designated healthcare provider to discuss the results of the pharmacogenetic test with the patient?** ***Can you elaborate on this?*** *(Enter only one answer)*	
1	Pharmacist
2	Treating physician in the hospital
3	General practitioner
4	Clinical geneticist
5	Other, namely..................
Explanation:	

**Room for remarks**
