# Supplementary figures and images for: Feasibility of Community Pharmacist-Initiated and Point-of-Care CYP2C19 Genotype-Guided De-Escalation of Oral P2Y12 Inhibitors
Source: Genes (Basel). 2023 Feb 25;14(3):578. doi: 10.3390/genes14030578 (PMC10048116; doi:10.3390/genes14030578)

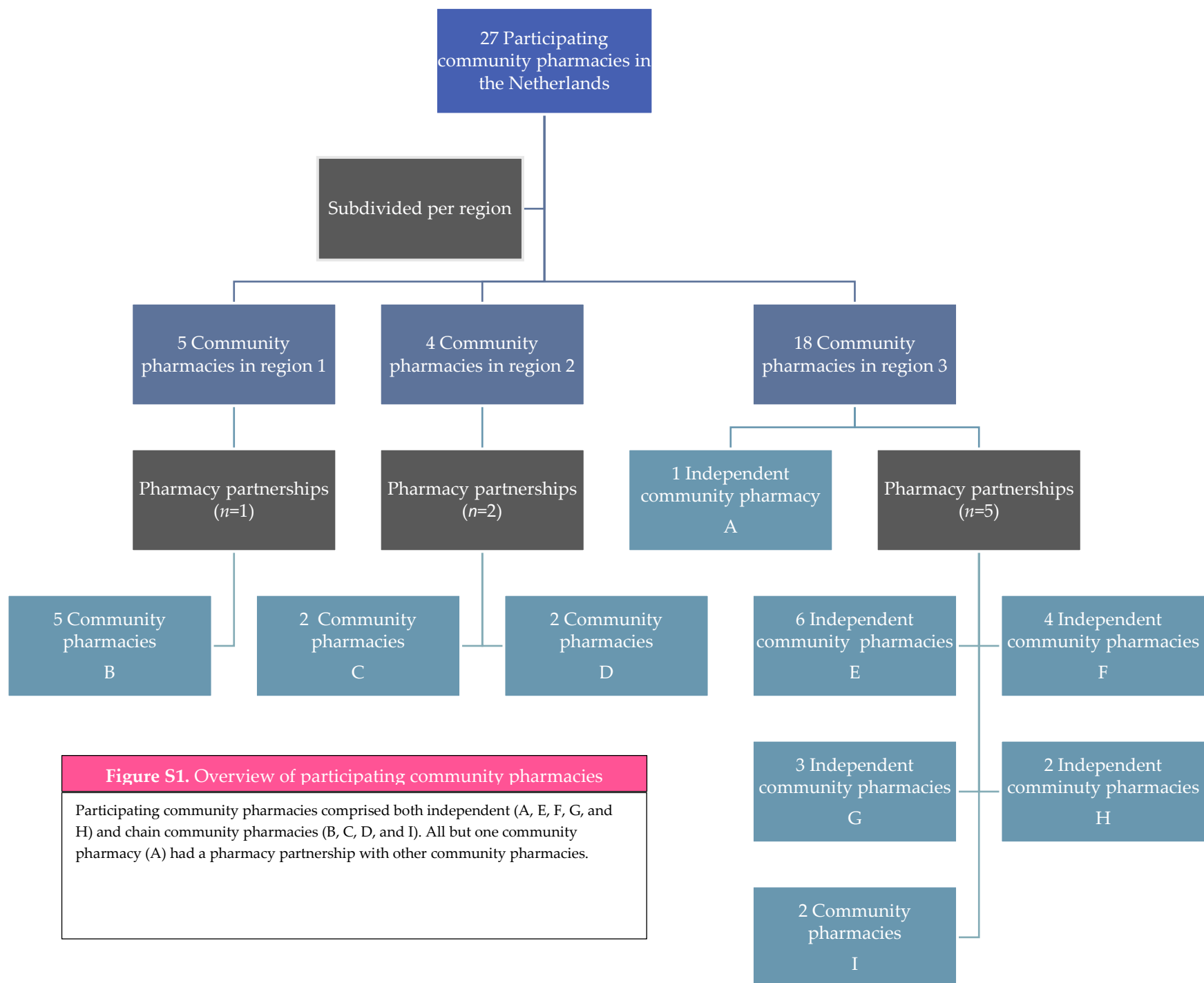

Supplement: Supplementary file 1 [file genes-14-00578-s001.zip › Figure S1 Overview of participating community pharmacies.pdf]

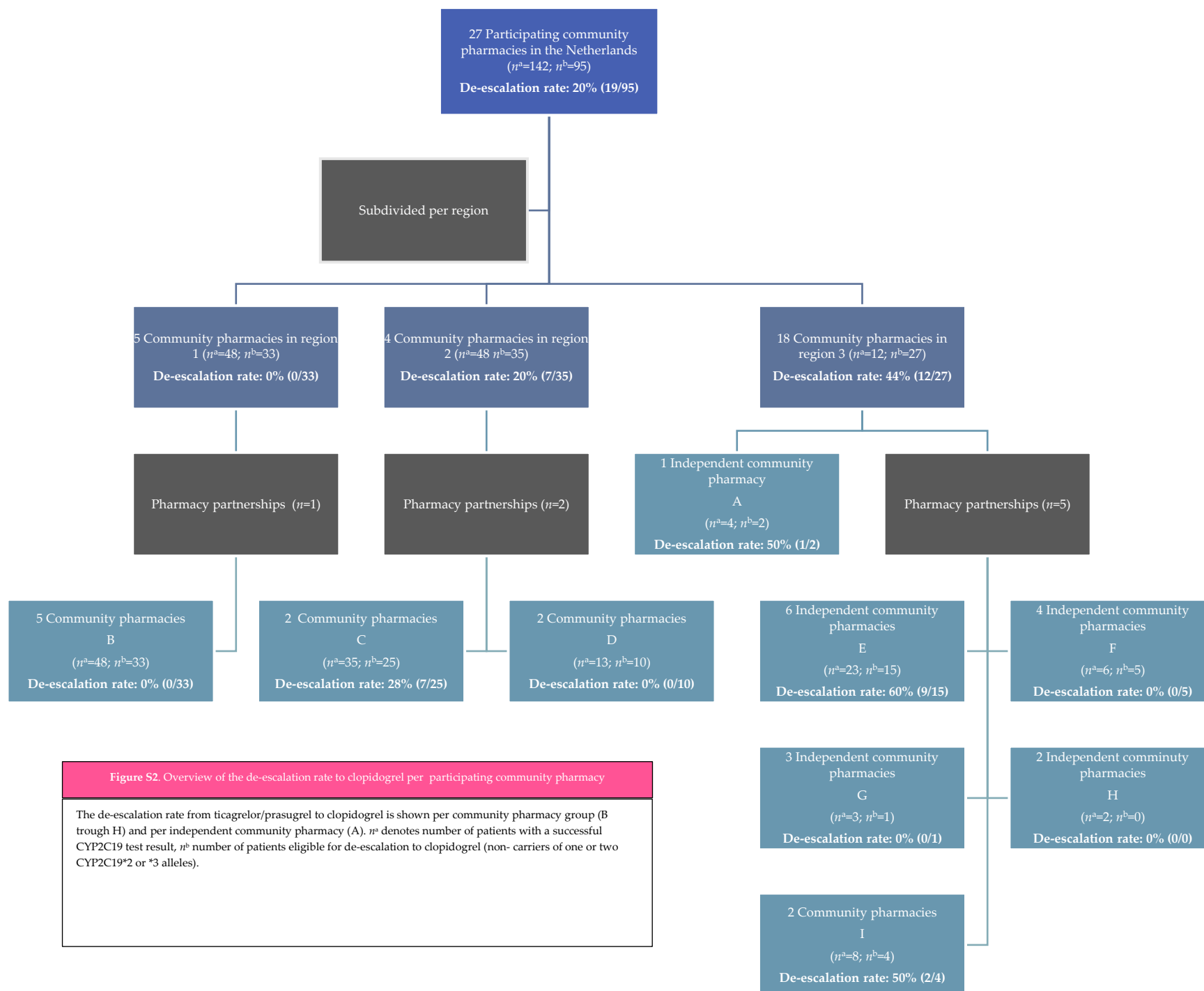

Supplement: Supplementary file 1 [file genes-14-00578-s001.zip › Figure S2 De-escalation rate to clopidogrel per community pharmacy group.pdf]
